# Supplementary material for: Preliminary Evaluation of Plasma circ_0009910, circ_0027478, and miR-1236-3p as Diagnostic and Prognostic Biomarkers in Hepatocellular Carcinoma
Source: Int J Mol Sci. 2025 May 19;26(10):4842. doi: 10.3390/ijms26104842 (PMC12111893; doi:10.3390/ijms26104842)
Supplement: Supplementary file 1 [file ijms-26-04842-s001.zip › ijms-3609033-supplementary.pdf]

Methods

The secondary structures of several circular RNAs, including hsa_circ_0027478, hsa_circ_0009910, and hsa_circ_0051732, were predicted using the RNAfold tool [1]. The RNAfold, which utilizes the minimum free energy (MFE) approach, was employed to generate the most thermodynamically stable structures for each circRNA. Following structural prediction, potential microRNA (miRNA) binding sites and RNA-binding protein (RBP) interactions were analyzed to identify possible regulatory interactions using circinteractome database [2].

Results

The secondary structures of hsa_circ_0027478, hsa_circ_0009910, and hsa_circ_0051732 were predicted using RNAfold, generating both minimum free energy (MFE) structures and centroid structures. The predicted structures highlight the stability and potential functional regions of these circular RNAs.

hsa_circ_0027478

The secondary structure of hsa_circ_0027478, derived from the NUP107 gene, was predicted with an optimal MFE structure of -251.90 kcal/mol and a centroid structure with an MFE of -196.90 kcal/mol. The structural analysis revealed an extended conformation with multiple stem-loop formations (**Figure 1**).

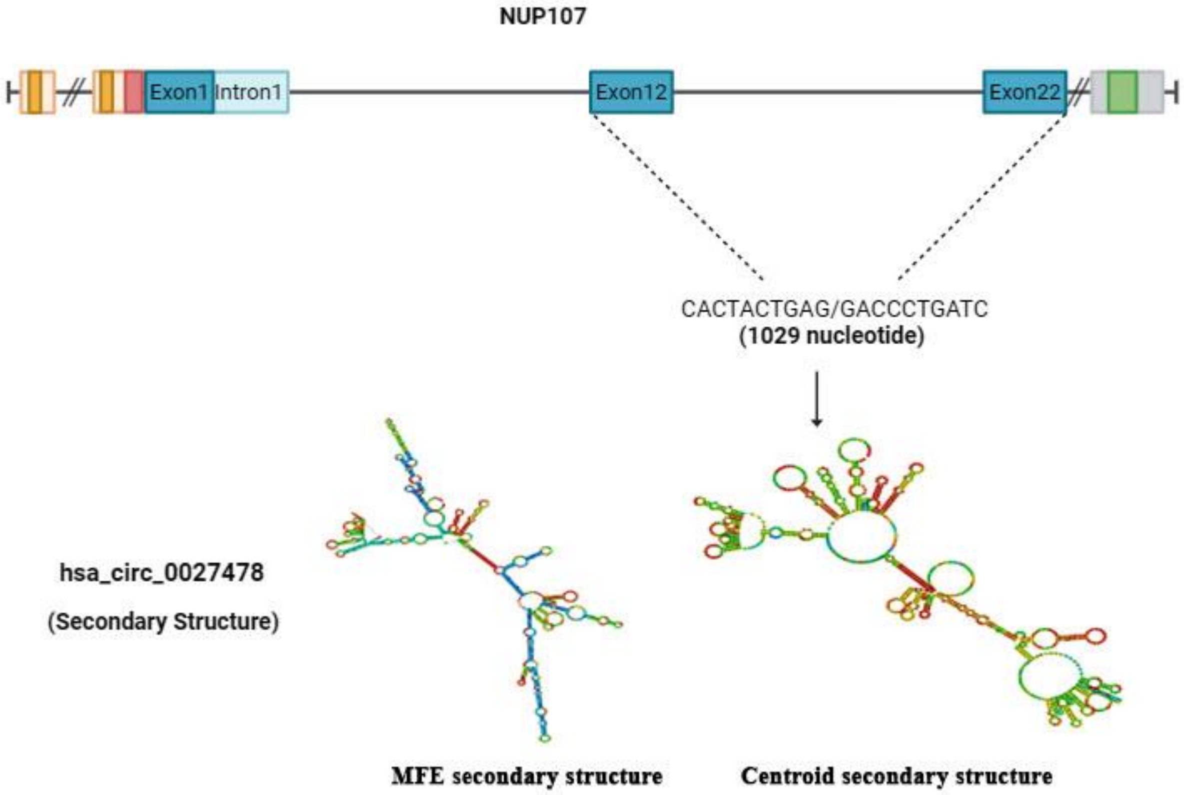

Suppl. Figure S1 Predicted Secondary Structure of hsa_circ_0027478

hsa_circ_0009910

The secondary structure of hsa_circ_0009910, originating from MFN2, displayed an optimal MFE of -77.50 kcal/mol, with the centroid structure showing an MFE of -56.90 kcal/mol. The predicted structure exhibits a central loop with multiple branching hairpins. The differences between the MFE and centroid structures suggest structural flexibility, which may influence its binding affinity for miRNAs or RNA-binding proteins (**Figure 2**).

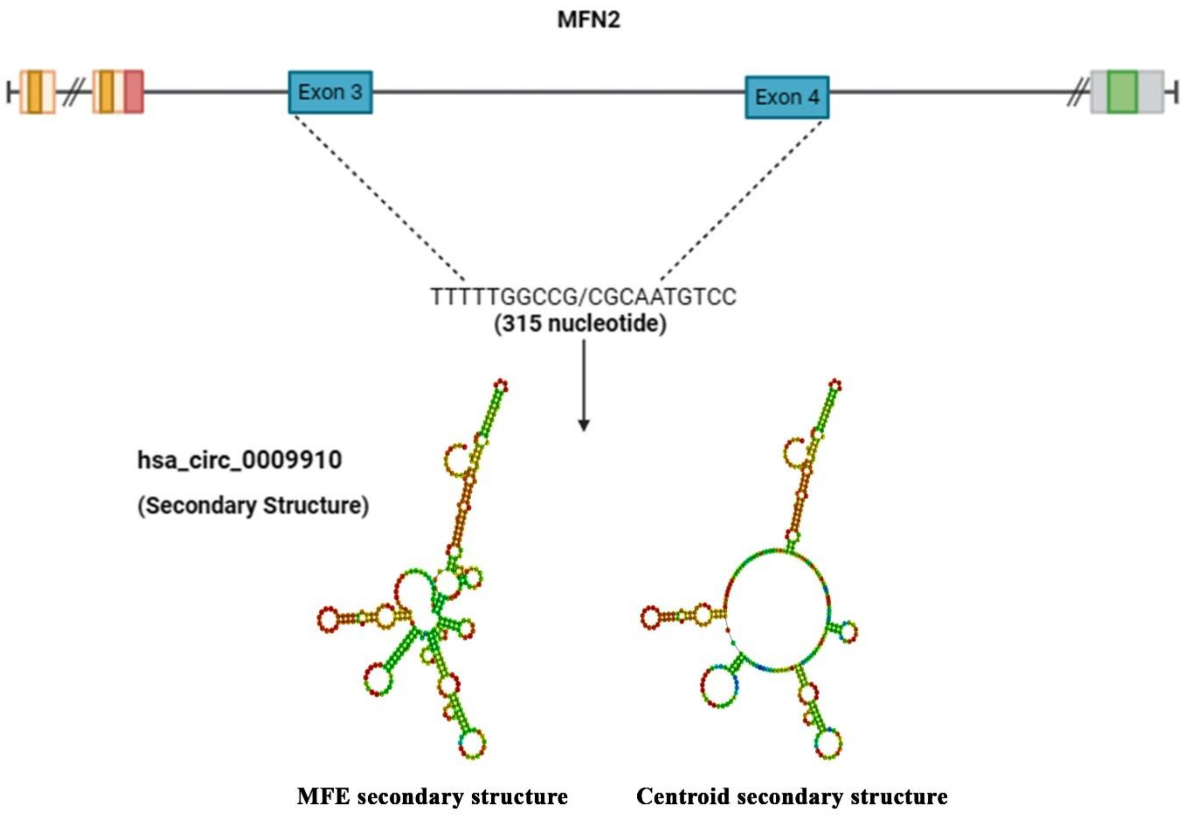

Suppl. Figure S2 Predicted Secondary Structure of hsa_circ_0009910

CircRNA-miRNA-RNA binding proteins Interaction Network

The network visualization reveals the intricate interactions between circular RNAs (circRNAs), microRNAs (miRNAs), and RNA-binding proteins (Figure 4). Three central circRNAs—hsa_circ_0009910, and hsa_circ_0027478—act as key hubs, each interacting with multiple miRNAs. hsa_circ_0027478 exhibits the highest connectivity with several miRNA and RNA binding proteins **(Figure 4, Supplementary tables)**. Several RNA-binding proteins, including HuR, and FMRP bind with the three microRNAs. Notably, **miR-1236 is highlighted in red, possibly signifying its distinct regulatory importance.**

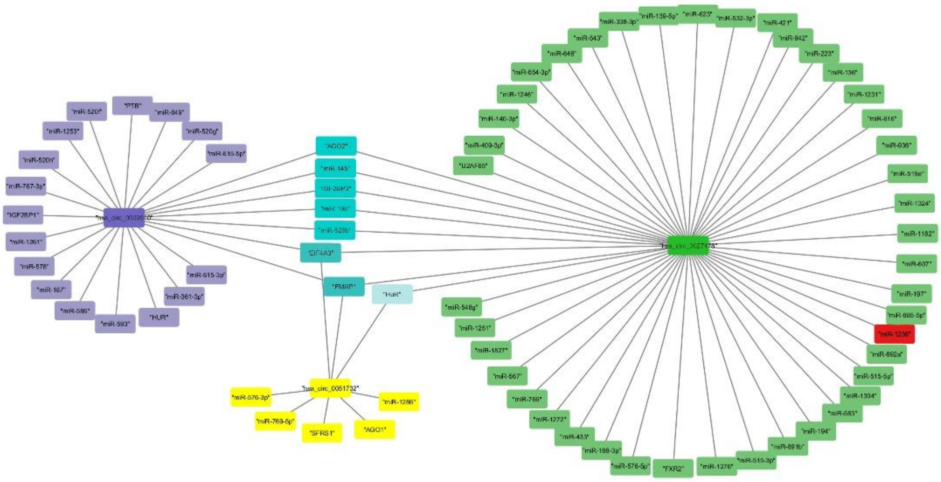

Suppl. Figure S3. CircRNA-miRNA-Protein Interaction Network

Reference

1- Lorenz, R., Bernhart, S.H., Höner zu Siederdissen, C. et al. ViennaRNA Package 2.0. Algorithms Mol Biol 6, 26 (2011). https://doi.org/10.1186/1748-7188-6-26

2- Dudekula, D. B., Panda, A. C., Grammatikakis, I., De, S., Abdelmohsen, K., & Gorospe, M. (2016). CircInteractome: A web tool for exploring circular RNAs and their interacting proteins and microRNAs. RNA Biology, 13(1), 34–42. https://doi.org/10.1080/15476286.2015.1128065
